# Eutrophication shifts microbial communities and life-history strategies in the Yangtze River Estuary

**DOI:** 10.3389/fmicb.2025.1650511

**Published:** 2025-09-15

**Authors:** Haizhou Li, Feng Zhao, Qunhui Yang, Lang Chen, Jin Zhou

**Affiliations:** ^1^East China Sea Fisheries Research Institute, Chinese Academy of Fishery Sciences, Shanghai, China; ^2^State Key Laboratory of Marine Geology, Tongji University, Shanghai, China

**Keywords:** Yangtze River Estuary, East China Sea, eutrophication, community assembly, life-history strategies

## Abstract

Marginal seas are increasingly impacted by anthropogenic activities, leading to widespread eutrophication, yet the responses of marine microbial communities remain poorly understood. We compared sediments from the highly eutrophic Yangtze River Estuary (YRE) and the oligotrophic East China Sea (ECS) to examine how eutrophication alters microbial abundance, community structure, assembly processes, functional profiles, and life-history strategies. Our results showed that YRE sediments harbored significantly higher microbial abundance (1.3 × 10^8^-1.1 × 10^9^ cells g^−1^ vs. 8.0 × 10^7^-7.1 × 10^8^ cells g^−1^), Chao1 richness (9,782–18,129 vs. 9,366–14,903), and Shannon diversity (6.19–7.47 vs. 6.05–7.07). Functional profiling revealed an enrichment of nitrogen- and carbon-cycling genes, human pathogens, and antibiotic-resistance genes in YRE. Life-history traits in YRE microbial communities showed higher average 16S rRNA gene copy numbers (median 2.75 vs. 2.56), greater codon usage bias (0.0181 vs. 0.0178), higher maximum predicted growth rates (0.1054 vs. 0.0951 h^−1^), larger genome sizes (5.59 vs. 5.46 Mb), higher GC content (56.43 vs. 55.83%) and increased transposase abundance (3.46 vs. 1.71%), collectively indicating a shift from *K*-strategists to *r*-strategists in the eutrophic environment. Neutral and null model analyses, and statistical analyses revealed that human activities, especially those altering water quality and chemistry, drive significant shifts in microbial community structure, function, and assembly processes, which in turn reshape microbial life-history strategies in estuarine benthic ecosystems.

## 1 Introduction

Microbes constitute the vast majority of organisms on Earth and exhibit immense taxonomic diversity and metabolic complexity (Chen et al., 2021; Osburn et al., 2024). This complexity can be distilled by grouping microorganisms based on their life-history strategies (Bao et al., 2024; Darling et al., 2012). Life-history strategies refer to suites of traits that tend to co-vary due to evolutionary or physiological trade-offs, with different strategies favored under varying environmental conditions. Life-history strategies framework allows for a direct connection between microbial traits and environmental regimes. Based on life-history theory, microbial ecologists have proposed several life-history trait-based strategies for environmental microbes, including the *r*- and *K*-strategies, the Competitor–Stress tolerator–Ruderal (C–S–R) model, the high yield–resource acquisition–stress tolerance (Y–A–S) framework, and the copiotrophic–oligotrophic strategy (Soler-Bistué et al., 2023; Couso et al., 2023; Malik et al., 2019).

In general, *r*- and *K*-strategies are often paralleled by the copiotroph–oligotroph classification, which classifies microorganisms based on their growth rates, nutrient utilization, and substrate preferences (Pianka, 1970; Yang et al., 2023). This conceptual model has been extensively used in environmental research to assess microbial community responses to anthropogenic disturbances (Yang et al., 2023; Yin et al., 2022). Microorganisms classified as *r*-strategists, or copiotrophs, typically thrive in resource-rich environments, exhibiting fast growth rates, low substrate affinity, and quick responses to organic matter inputs and nutrient pulses (Zhu and Dai, 2024). In contrast, oligotrophs or *K*-strategists are better suited to nutrient-scarce environments, characterized by slow growth, high substrate affinity, and an enhanced capacity to degrade complex or refractory organic compounds (Yin et al., 2024; Grime, 1977). *r*- and *K*-strategies reflect a fundamental ecological trade-off between growth rate and resource-use efficiency (Ferenci, 2016).

Advancements in metagenomic sequencing now allow for the detailed examination of microbial community-level traits, facilitating the inference of life-history strategies across ecosystems. One of the most widely used traits for differentiating *r*- and *K*-strategists is the copy number of ribosomal RNA (rRNA) genes (Piton et al., 2023; Roller et al., 2016). *r*-strategists tend to harbor multiple rRNA operons, enabling rapid protein synthesis and fast population expansion in nutrient-enriched environments (Osburn et al., 2024; Vieira-Silva and Rocha, 2010). These microbes also show strong codon usage bias in ribosomal genes, reflecting a selective pressure for translational efficiency. In contrast, *K*-strategists generally maintain fewer rRNA operons, a trait associated with slower growth rates and streamlined genomes (Giovannoni et al., 2014; Shenhav and Zeevi, 2020). Genome streamlining reflects an evolutionary trade-off favoring resource efficiency, enabling oligotrophic microorganisms to sustain growth and reproduction with minimal energy input (Giovannoni et al., 2014).

Estuarine eutrophication, which is caused mainly by human activities, is widely distributed and is a major challenge for global environmental protection (Zhou et al., 2023; Wang et al., 2012; Chen J. et al., 2019). In China, the Yangtze River Estuary (YRE) is facing severe eutrophication. As the largest river in the country, the Yangtze River flows through densely populated urban, industrial, and agricultural regions, making it a major accumulation zone for anthropogenic contaminants (ACs; Wen et al., 2024). Over the past decade, the annual transport fluxes of total organic carbon (TOC), total nitrogen (TN), and total phosphorus (TP) from the Yangtze River to the East China Sea (ECS) have increased significantly, reaching 5.00, 1.69, and 0.11 Tg/yr, respectively (Hua et al., 2019; Bianchi and Allison, 2009). These excessive nutrient loads have caused severe eutrophication, promoting noxious and toxic algal blooms (Wen et al., 2024) and leading to increasingly frequent hypoxic events in the YRE, which further exacerbate the expansion of the oxygen minimum zone (OMZ; Dai et al., 2011). Moreover, extensive studies have shown that YRE sediments are heavily contaminated with diverse ACs (Chai et al., 2006; Liu et al., 2021), including antibiotics (Guo et al., 2019), heavy metals (Cao et al., 2015), polycyclic aromatic hydrocarbons (PAHs; Guo et al., 2007), and endocrine-disrupting chemicals (Bian et al., 2010).

Despite these findings, the mechanistic links between human-induced changes in overlying water conditions and benthic microbial life-history strategies remain poorly understood (Bao et al., 2024). Human activities directly affect environmental factors in overlying water, which in turn influence the trophic status and microbial communities reflected in sediments. In this context, changes in overlying water environmental factors act as the primary drivers, whereas the sediment trophic status and microbial communities reflect the outcomes of these changes. Therefore, analyzing the relationship between environmental factors in the overlying water and microbial life-history strategies in sediments provides a more integrated perspective on the ecological impacts of eutrophication.

**Figure 1:**
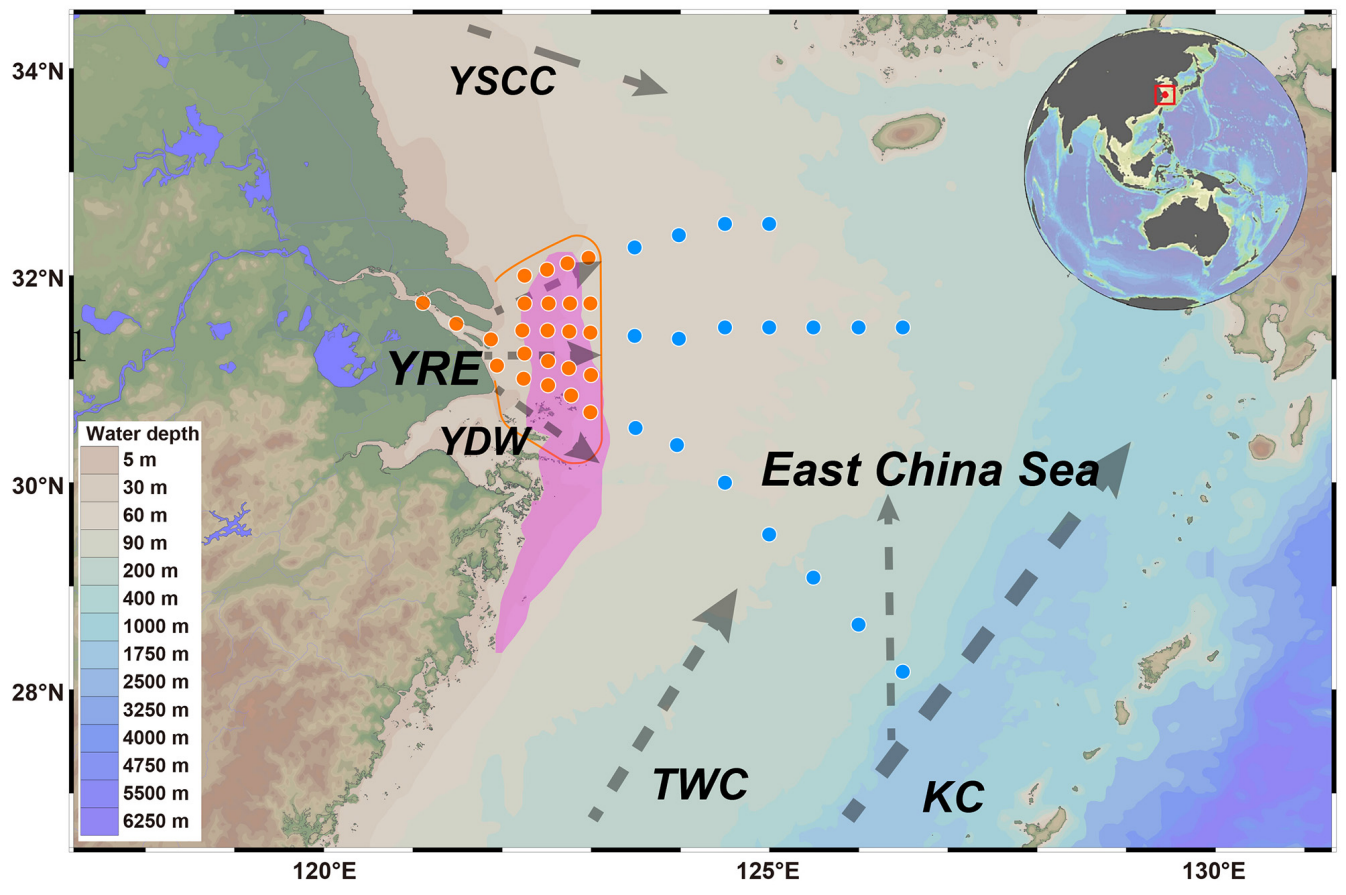
The locations of the overlying water and sediment sample collection sites in the Yangtze River Estuary (orange circles) and East China Sea (blue circles) are displayed on the map. The purple polygon indicates the OMZ. The dotted gray line represents the currents that flow throughout the year, including the Yangtze diluted water (YDW), Taiwan warm current (TWC), Kuroshio current (KC), and Yellow Sea coastal current (YSCC). The orange line indicates that the main dispersion of modern Yangtze water is constrained west of 123°E.

Accordingly, we collected sediment samples from the highly eutrophic Yangtze River Estuary (YRE) and the adjacent, relatively undisturbed East China Sea (ECS; Figure 1), allowing for a comprehensive and robust evaluation of human-driven nutrient pollution impacts on the marine microbial biosphere. Using a combination of 16S rRNA amplicon sequencing and shotgun metagenomics, we characterized microbial community composition, diversity, assembly processes, functional profiles, prevalence of pathogenic bacteria, abundance and potential spread of antibiotic-resistance bacteria, as well as microbial life-history strategies within estuarine sediments. Our findings reveal that human activities, especially those altering water quality and chemistry, drive significant shifts in microbial community structure, function, and assembly processes, which in turn reshape microbial life-history strategies in estuarine benthic ecosystems.

## 2 Materials and methods

### 2.1 Study area and physicochemical analysis

Based on long-term historical records (Lu et al., 2017) and data from the NOAA World Ocean Atlas (https://repository.library.noaa.gov/view/noaa/60601; Garcia et al., 2023), dissolved oxygen (DO) levels in the Yangtze River Estuary (YRE) are generally lower during summer and autumn compared to winter and spring, with the most pronounced oxygen minimum zone (OMZ) typically occurring in July. Accordingly, both overlying water and sediment samples were collected in July 2024 (Figure 1). Overlying water samples, taken from 1 meter above the seafloor, were collected using an SBE−911 Plus CTD profiling system. At each site, surface sediments (0–10 cm) were retrieved using a 150 mm diameter gravity corer. All samples were placed in sterile bags, kept in the dark, and transported on dry ice before storage at −80 °C.

Water quality parameters—including ammonia, nitrite, nitrate, phosphate, silicate, chlorophyll a, dissolved oxygen, and salinity—were measured according to the *Environmental Quality Standards for Seawater* (Ministry of Ecology and Environment of the People's Republic of China, GB 3097–1997). Sediment total organic carbon (TOC) and total nitrogen (TN) were analyzed using a Multi N/C 3100 TOC analyzer (Analytik Jena, Germany).

Each environmental variable at each site was measured in triplicate, and the mean of the three replicates (*n* = 3) was used to represent the value for that sampling point. All results for the YRE and ECS were thus analyzed at the individual sample level rather than as region-level averages. Specifically, each sample from the YRE (*n* = 24) and ECS (*n* = 18) was treated as an independent data point in the comparative analyses between the two regions. The same sample-level data processing approach was consistently applied across all subsequent analyses in this study.

### 2.2 Trophic status of the overlying water samples

Trophic status of the overlying water was estimated using the Trophic Index (TRIX; Vollenweider et al., 1998). This index is a linear combination of chlorophyll-a concentration, nutrient (nitrogen and phosphorus) log scale concentrations, and dissolved oxygen levels. This combination of four state variables describes primary production (chlorophyll-a and oxygen), and nutritional conditions (nitrogen and phosphorus). The TRIX index was calculated as follows:


(1) $$\begin{array}{l} \text{TRIX}=\left(Log10\left[\text{Chl}-\text{a}\cdot \text{aD\%O}\cdot \text{DIN}\cdot \text{P}\right]+\text{x}\right)/\text{m} \end{array}$$

Where, *P* = soluble reactive phosphorus (μg/L); DIN = dissolved inorganic nitrogen (μg/L); Chl-*a* = chlorophyll-*a* concentration, as μg/L; aD%O = absolute % of oxygen deviation from saturation; *x* and *m* = scale coefficients were introduced to fix the lower limit value of the index and the extension of related trophic scale from 0 to 10. This index was calculated for the whole area and then for each particular zone. Here, *x* = 1.5 and *m* = 1.2. TRIX ≤ 4: oligotrophic; 4 <TRIX ≤ 5: mesotrophic; TRIX > 5: eutrophic (Yan et al., 2022).

### 2.3 Eutrophication status of the sediment samples

The organic index (OI) and organic nitrogen (ON) are key indicators used to evaluate the nutrient status of sediment samples (Yang et al., 2017). The classification criteria for OI and ON levels are summarized in Table 1. Their values were calculated using the following equations (Li et al., 2024):


(2) $$\begin{array}{l} \text{ON}=\text{TN}\times 95\% \end{array}$$

(3) $$\begin{array}{l} \text{OI}=\text{TOC}\times \text{ON} \end{array}$$

**Table 1:** Evaluation criteria for OIs and ONs in sediments (Li et al., 2024).

**OI (%)**	**ON (%)**	**Pollution status**	**Level**
<0.05	<0.0033	Uncontaminated	I
0.05–0.20	0.0033–0. 66%	Slightly eutrophication	II
0.20–0.50	0.66–0.13%	Moderately eutrophication	III
≥0.50	>0.13	Heavily eutrophication	IV

### 2.4 Sediment cell counts

Cell enumeration using the catalyzed reporter deposition fluorescence *in situ* hybridization (CARD-FISH) protocol followed procedures described in our previous study (Li et al., 2023). The probe-labeling peroxidases were EUB338 (bacteria: 5′-GCW GCC WCC CGT AGG WGT-3′) and Arch915 (archaea: 5′-GTG CTC CCC CGC CAA TTC CT-3′; Li et al., 2015). Probe NON338 (5′-ACT CCT ACG GGA GGC AGC-3′) was used as a control (Wallner et al., 1993). Paraformaldehyde (4 mL; 4% solution in 100 mM phosphate-buffered saline) was added to autoclaved 15-mL plastic tubes. Then, 1 g sediment was added to each tube, bringing the volume to 5 mL total. Two replicate tubes were prepared for cell counts for each sample. Preserved samples were stored at 4 °C for onshore analysis. One milliliter of the fixed sample slurry was used 40 cycles of sonication in the quantification procedure to better release cells attached to the sediments (Morono et al., 2013). After that, 100 μL of dispersed samples was diluted in 20 mL of Milli-Q filtered water and was filtered on polycarbonate filters, Next, 0.1% low melting point agarose was dripped onto the filters and dried at 46 °C in an incubator. The microbes were permeabilized using 15 μg/mL proteinase K. Then, 3% H_2_O_2_ was used to inactivate the endogenous peroxidases. For hybridization, filters were placed in a tube and mixed with 500 μL hybridization solution [10% dextran sulfate, 2% blocking reagent (Roche, Germany), 0.1% (w/v) sodium dodecyl sulfate, 20 mM Tris–HCl (pH 8.0), 0.9 M NaCl and formamide] and 1 μL of probe working solution (final concentration, 0.028 μM; Eickhorst and Tippkötter, 2008). Microorganisms were hybridized for at least 60 min on a rotor at 46 °C, and then the filters were washed twice using washing solution [Eickhorst and Tippkötter, 2008; 0.01% SDS, 5 mM EDTA (pH 8.0), 20 mM Tris–HCl (pH 8.0) and 3 mM NaCl] at 48 °C for 20 min. After washing, filters were mixed with 1,000 μL of amplification solution [0.0015% H_2_O_2_, 1 × PBS (pH 7.4), 0.1% (w/v) blocking reagent] and 1 μL of Alexa 488-labeled tyramides (Life Technologies™, Thermo Fisher, USA). The probes were incubated at 46 °C in amplification solution for at least 30 min in the dark. For second hybridizations, the first probe-labeling peroxidase was inactivated by incubating the filter sections in 0.01 M HCl for 10 min at room temperature and washing the sections with 50 mL of Milli-Q water. Next, the CARD-FISH protocol was repeated two times with the same filter sections by using different probes. The second hybridization was performed using Alexa 647-labeled tyramides (Life Technologies, Thermo Fisher, USA). Finally, all microorganisms were mounted with ProLong Gold Antifade reagent (Life Technologies, Carlsbad, California, USA). Cell counting was performed in triplicate using ImageJ, and the cell abundance were expressed as the mean (*n* = 3).

### 2.5 16S rRNA amplicon high-throughput sequencing and analysis in the sediment samples

At each sampling site, three sediment subsamples were collected and homogenized prior to DNA extraction and sequencing. The V4 hypervariable region of the 16S rRNA gene was amplified by PCR using universal bacterial and archaeal primers 515F and 806R, following the Earth Microbiome Project protocol (Hoshino et al., 2020). Purified amplicons were pooled in equimolar concentrations and sequenced using the Illumina MiSeq platform (2 × 300 bp, Illumina, USA). Raw FASTQ files were demultiplexed, quality-trimmed using Trimmomatic, and merged with FLASH. Operational taxonomic units (OTUs) were clustered at 97% sequence similarity using UPARSE, and chimeric sequences were identified and removed with UCHIME. Alpha diversity metrics—including observed OTU richness, Chao1, Shannon, and Simpson indices—were calculated using Mothur. Taxonomic classification was performed against the SILVA reference database (version 138). To estimate microbial coexistence in different regions, metacommunity co-occurrence networks were established for YRE and ECS. To reduce the influence of rare OTUs in the dataset, we retained only the top 1,000 OTUs ranked by relative abundance. Robust correlations with Spearman's correlation coefficients (ρ) > 0.6 and false discovery rate-corrected *P* < 0.001 were used to construct networks. Each node represents one OTU, and each edge represents a strong and significant correlation between two nodes.

### 2.6 Niche breadth

Niche breadth was used to quantify habitat specialization (Levins, 1968). The formula is as follows:


(4) $$\begin{array}{l} B_{j}=\frac{1}{\sum_{i=1}^{N}P_{ij}^{2}} \end{array}$$

Where, *B*_*j*_ represents the habitat niche breadth of OTUj in a metacommunity; *P*_*ij*_ is the proportion of OTUj at a given site *i*; and *N* is the total number of sites. The average *B* value across all the OTUs in a community is referred to as the community-level habitat niche breadth (*Bcom*; Jiao et al., 2020).

### 2.7 Neutral community model and null model

To assess the role of stochastic processes in shaping microbial communities, we employed the Neutral Community Model (NCM) as proposed by Sloan et al. (2006). This model evaluates the relationship between the frequency at which individual taxa are detected in local communities and their overall abundance within a broader metacommunity. According to the NCM, taxa with high relative abundance are more likely to disperse randomly and become widespread, whereas low-abundance taxa are more vulnerable to stochastic loss through ecological drift. The estimated migration rate (*m*) serves as a proxy for dispersal limitation, representing the likelihood that a lost individual in a local population is replaced through immigration from the metacommunity (Zhou and Ning, 2017). The model is mathematically represented as:


(5) $$\begin{array}{l} Freq_{i}=1-I\left(1/N|N^{*}m^{*}p_{i},N^{*}m^{*}\left(1-p_{i}\right)\right) \end{array}$$

Where, *Freq*_*i*_ is the occurrence frequency of taxon *i* across communities; *N* is the number of individuals per community; *m* is the estimated migration rate; *p*_*i*_ is the average relative abundance of taxon *i* across communities; and *I*() is the cumulative density function of the beta distribution. Higher *Nm* values imply that microbial communities are less limited by dispersal.

In parallel, a null model framework following Stegen et al. (2012) was used to disentangle the ecological processes governing community assembly, including selection, dispersal limitation, homogenizing dispersal, and ecological drift. To quantify taxonomic and phylogenetic turnover, we calculated the β-nearest taxon index (βNTI) and the Raup–Crick metric based on Bray–Curtis dissimilarities. A |βNTI| > 2 implies significant phylogenetic turnover driven by deterministic selection: βNTI > +2 suggests variable selection across environments, while βNTI <−2 indicates consistent selection pressures (homogeneous selection). For comparisons not dominated by selection (|βNTI| ≤ 2), the RC_Bray_ was used to distinguish dispersal-related mechanisms. RC_Bray_ > 0.95 signifies dispersal limitation, RC_Bray_ <−0.95 indicates homogenizing dispersal, and values between −0.95 and +0.95 suggest an “undominated” process, potentially involving weak selection, drift, or diversification (Dini-Andreote et al., 2015).

### 2.8 Metagenomic sequencing and functional annotation in the sediment samples

Metagenomic libraries were constructed using the Nextera DNA Library Prep Kit (Illumina), followed by paired-end sequencing (2 × 150 bp) on the Illumina HiSeq 3000 platform. Adapter sequences and barcodes were removed using Trimmomatic (Reysenbach et al., 2020), and the resulting high-quality reads were interleaved. Both interleaved and singleton reads were assembled into contigs using MEGAHIT (Li et al., 2016), a *de novo* assembler optimized for metagenomic data. To explore functional gene profiles, open reading frames were predicted from assembled contigs using Prodigal, and gene functions were annotated against the Kyoto Encyclopedia of Genes and Genomes (KEGG) database via GhostKOALA (Kanehisa et al., 2016). Additional functional annotations were obtained using multiple specialized databases, including eggNOG (Huerta-Cepas et al., 2015) for orthologous groups, PHI-base (Urban et al., 2021) for host-pathogen interactions, ARDB (Liu and Pop, 2009) and ResFams (Gibson et al., 2015) for antibiotic resistance genes. The abundance of key functional genes was normalized and expressed as reads per million mapped reads (RPM).

### 2.9 Determination of life-history traits in the sediment samples

For each sample, we calculated a set of traits from metagenomic data to shed light on the microbial life-history strategies. Estimates of the average 16S rRNA gene copy number and genome size were carried out based on the workflow proposed by Chen et al. (2021). Genome counts were inferred from the mean sequencing coverage of 35 universal single-copy marker genes. The 16S rRNA gene copy number per genome was calculated by dividing the total coverage of 16S rRNA genes by the estimated genome count (Pereira-Flores et al., 2019). Similarly, average genome size was derived by dividing the total assembled base pairs by the estimated number of genomes. To further assess microbial growth strategies, codon usage bias (CUB) in ribosomal genes was analyzed following the approach described by Vieira-Silva & Rocha (Vieira-Silva and Rocha, 2010; available at https://galaxy.pasteur.fr/?tool_id=toolshed.pasteur.fr%2Frepos%2Fkhillion%2Fgrowthpred%2Fgrowthpred%2F1.07&version=1.07&__identifer=unl8nld8ngn). Ribosomal proteins were identified via BLAST alignment against reference datasets of fully sequenced genomes. Codon usage bias for each ribosomal gene was quantified using the effective number of codons (ENC′), and the community-level codon bias was represented as the inverse of the mean ENC′ across all identified ribosomal genes (Huerta-Cepas et al., 2015). The predicted minimum generation time was inferred from codon usage patterns, and the maximum potential growth rate was estimated as the inverse of this generation time (h^−1^; Urban et al., 2021). Additionally, the GC content of quality-filtered metagenomic reads was calculated following the protocol outlined by Barberán et al. (2012). While our study utilizes 16S rRNA gene copy number and codon usage bias (CUB) as proxies to infer microbial life-history strategies, we acknowledge several inherent limitations associated with these genomic traits. First, intra-genomic variation in rRNA operon copy number may lead to over- or underestimation of the actual growth potential of some taxa, especially those with highly plastic genomes. Second, codon usage bias, although widely associated with translational efficiency and growth rate, may also be shaped by other factors such as phylogenetic constraints, mutational bias, genome size, and GC content. Therefore, these traits should be interpreted as indicative but not definitive markers of life-history strategies. Despite these caveats, when analyzed in aggregate across communities, these proxies still offer valuable ecological insights into microbial adaptations under eutrophication pressure.

### 2.10 Data available

The 16S rRNA amplicon and metagenomic sequencing data have been deposited in the NCBI Sequence Read Archive under accession numbers PRJNA1198359 and PRJNA1218293, respectively.

## 3 Results

### 3.1 Assessment of trophic status in overlying waters

**Figure 2:**
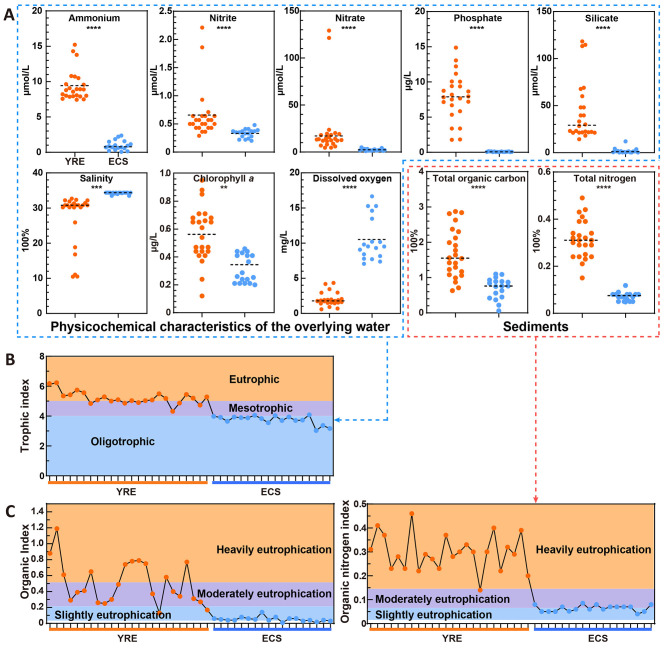
**(A)** Differences in environmental variables in overlying water samples (ammonium, nitrite, nitrate, phosphate, silicate, salinity, chlorophyll *a*, and dissolved oxygen) and sediment samples (TOC and TN) between the YRE (orange circles) and ECS (blue circles). Statistical comparisons were performed using the Wilcoxon rank-sum test with FDR correction (Benjamini–Hochberg method) and asterisks indicate significant differences between YRE and ECS samples (**P < 0.01; ***P < 0.001; ****P < 0.0001). **(B)** Trophic status of overlying water. Colors indicate trophic status levels: blue = oligotrophic, purple = mesotrophic, orange = eutrophic. **(C)** Contaminated assessment of the organic index and organic nitrogen index in the YRE and ECS sediments. Colors indicate contamination levels: white = uncontaminated, blue = uncontaminated to moderately contaminated, purple = moderate contamination, orange = heavy contamination.

To assess the impact of estuarine eutrophication on microbial communities, we first characterized the physicochemical properties of the overlying water in the YRE and ECS. Representative samples were collected from 24 YRE sites and 18 ECS sites (Figure 1). The sites covered a wide range of horizontal distances (9–865 km). In the YRE, the concentrations of ammonia, nitrite, nitrate, phosphate, silicate, chlorophyll *a*, and DO in the overlying water were 7.43–15.21 μmol/L, 0.29–2.21 μmol/L, 1.29–129.21 μmol/L, 0.05–3.19 μmol/L, 14.27–118.29 μmol/L, 0.12–0.95 μg/L, and 0.59–4.34 mg/L, respectively. In contrast, the ECS exhibited substantially lower values, with the concentrations of ammonia, nitrite, nitrate, phosphate, silicate, chlorophyll *a*, DO and salinity were 0.07–2.36 μmol/L, 0.20–0.48 μmol/L, 1.02–4.97 μmol/L, 0.02–0.33 μmol/L, 0.25–12.06 μmol/L, 0.2–0.65 μg/L, and 3.0–10.4 mg/L, respectively (Figure 2A, Supplementary Dataset 1A). Moreover, most YRE monitoring sites were affected by low-oxygen conditions (DO <2 mg/L), whereas no hypoxic waters were observed across the ECS sites.

Correspondingly, the trophic status assessed by the TRIX further confirmed these differences (Figure 2B, Supplementary Dataset 1A). The TRIX values for YRE sites ranged from 4.32 to 6.23, indicating a state of eutrophic estuary. In contrast, the ECS sites exhibited lower TRIX values ranging from 3.03 to 4.08, indicating a state of oligotrophic condition (Figure 2B).

### 3.2 Sediment eutrophication status

In the YRE sediments, the concentrations of TOC and TN ranged from 0.63 to 2.87% and 0.15 to 0.49%, respectively. In the ECS sediments, the TOC and TN contents ranged from 0.06 to 1.10% and 0.05 to 0.20%, respectively (Figure 2C, Supplementary Dataset 1B). There was a significant increase in the TOC and TN contents in the YRE relative to those in the ECS (*P* < 0.0001, Wilcoxon Rank-Sum Test). The eutrophication status was assessed on the basis of the OI and ON (Table 1; see Methods for further details). The results revealed that the OI values in the YRE and ECS ranged from 0.12 to 1.19 and 0.03 to 0.14, respectively (Figure 2C, Supplementary Dataset 1B). The ON values in the YRE and ECS ranged from 0.14 to 0.46 and 0.05 to 0.19, respectively. The OI and ON indices indicate that most YRE sites were classified as moderately to heavily eutrophic condition, in contrast to the ECS sites, which were characterized by slight to moderate eutrophication (Figure 2C, Table 1).

### 3.3 Microbial community abundance, richness, diversity, and composition in sediments

**Figure 3:**
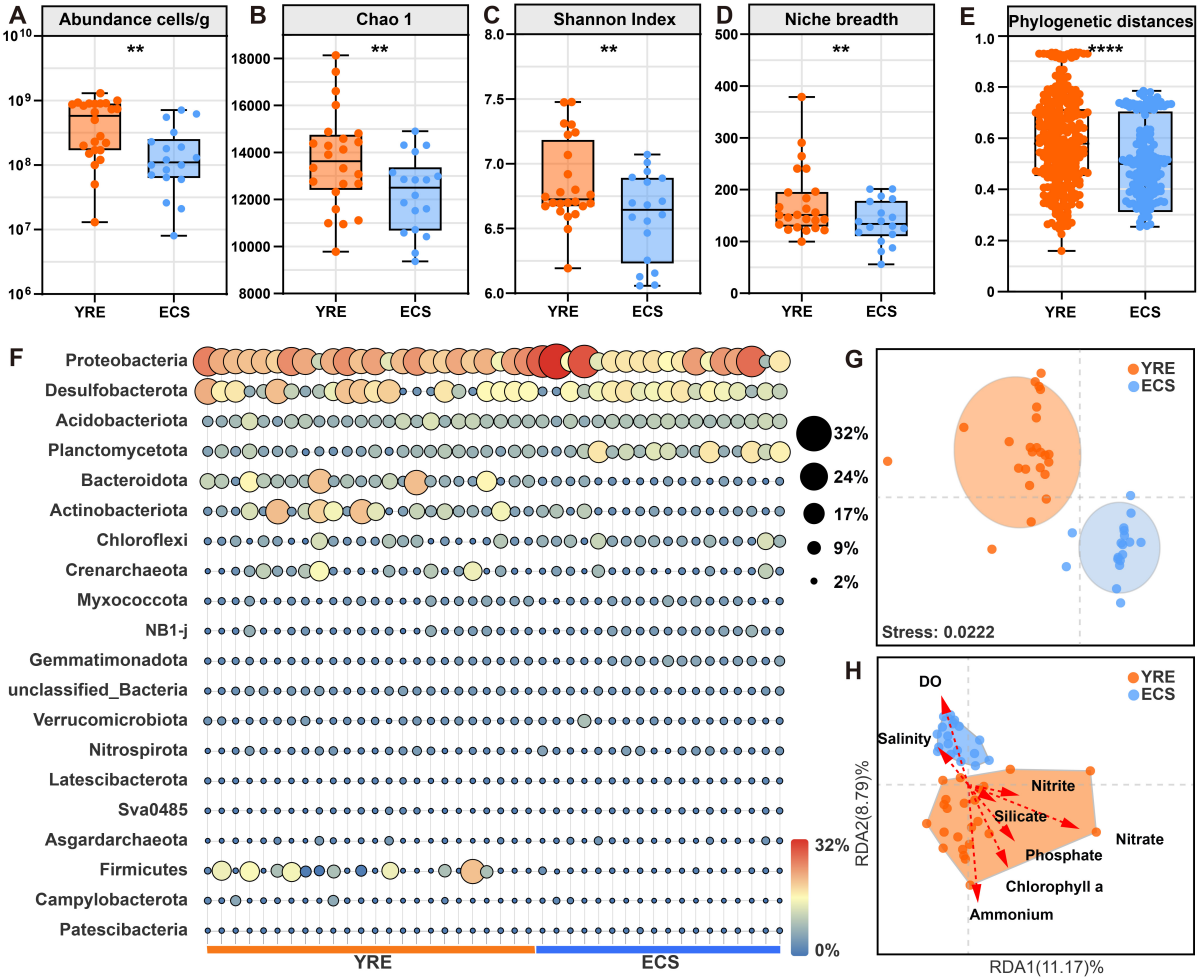
**(A–E)** Microbial abundance, Chao1 richness estimates, the Shannon index, niche breadth and phylogenetic distances were investigated in the YRE (orange circles) and ECS (blue circles) sediments based on amplicon data. Statistical comparisons were performed using the Wilcoxon rank-sum test with FDR correction (Benjamini–Hochberg method) and asterisks indicate significant differences between YRE and ECS samples (**P < 0.01; ****P < 0.0001). **(F)** Relative abundance of the most abundant microbes across sites at the phylum level in sediment samples. **(G)** Similarity of microbial community composition visualized by two-dimensional (2D) non-metric multidimensional scaling (NMDS) between the YRE and ECS. **(H)** Distance-based redundancy analysis (db-RDA), also visualized through 2D NMDS, illustrated microbial community similarity when environmental factors were used as constraining variables, with these factors represented as arrows on the ordination. The length of each arrow reflects the multiple partial correlations of the variable to the RDA axes, indicating the contribution of that variable to the explained similarity within the communities.

CARD–FISH analysis revealed that the microbial abundance varied between 1.3 × 10^8^ and 1.1 × 10^9^ cells g^−1^ in the YRE sediments and between 8.0 × 10^7^ and 7.1 × 10^8^ cells g^−1^ in the ECS sediments (Figure 3A, Supplementary Dataset 2A). Statistical comparison revealed that microbial populations in the YRE sediments were significantly higher than those in the ECS samples (*P* < 0.01, Wilcoxon Rank-Sum Test).

Using universal bacterial and archaeal 16S rRNA gene primers, Illumina sequencing of sediment genomic DNA yielded 4,828,964 and 3,050,297 high-quality reads for YRE and ECS samples, respectively (Supplementary Dataset 2A), which clustered into 48,545 operational taxonomic units (OTUs) at 97% similarity (Supplementary Dataset 2B). In YRE sediments, Chao1 richness estimates ranged from 9,782 to 18,129 OTUs, with Shannon diversity indices between 6.19 and 7.47. By comparison, ECS samples exhibited Chao1 values of 9,366 to 14,903 and Shannon indices from 6.05 to 7.07 (Figures 3B, C). Analysis of variance indicated that both Chao1 richness and Shannon diversity were significantly higher in YRE sediments than in ECS samples (*P* < 0.01 for both, Wilcoxon Rank-Sum Test). Additionally, the community-level habitat niche breadth (*Bcom*; Figure 3D) and phylogenetic distance (Figure 3E) were greater in YRE microbial communities relative to those from the ECS.

We constructed a detailed prokaryotic community profile spanning taxonomic levels from phylum to genus (Supplementary Dataset 2). At phylum level, 88 phyla and 85 phyla were identified in YRE and ECS benthic ecosystems, respectively. The top 10 phyla accounted for 76.43% of total sequences, aligning well with the typical abundance-range patterns observed in microbial ecology. In the YRE, the major phyla were Proteobacteria (20.96%), Desulfobacterota (13.22%), Acidobacteriota (8.71%), Planctomycetota (7.35%), Bacteroidota (6.30%), Actinobacteriota (5.21%), Crenarchaeota (5.06%), Chloroflexi (4.49%), Myxococcota (3.30%), NB1-j (2.81%; Figure 3F, Supplementary Dataset 2C). In the ECS, the 10 most prevalent phyla were Proteobacteria (20.16%), Desulfobacterota (13.20%), Acidobacteriota (9.26%), Planctomycetota (7.93%), Chloroflexi (6.24%), Actinobacteriota (4.64%), Gemmatimonadota (4.07%), NB1-j (3.75%), Bacteroidota (3.55%), Myxococcota (3.44%). To estimate microbial coexistence in different regions, metacommunity co-occurrence networks were established for YRE and ECS (Supplementary Figure S1).

**Figure 4:**
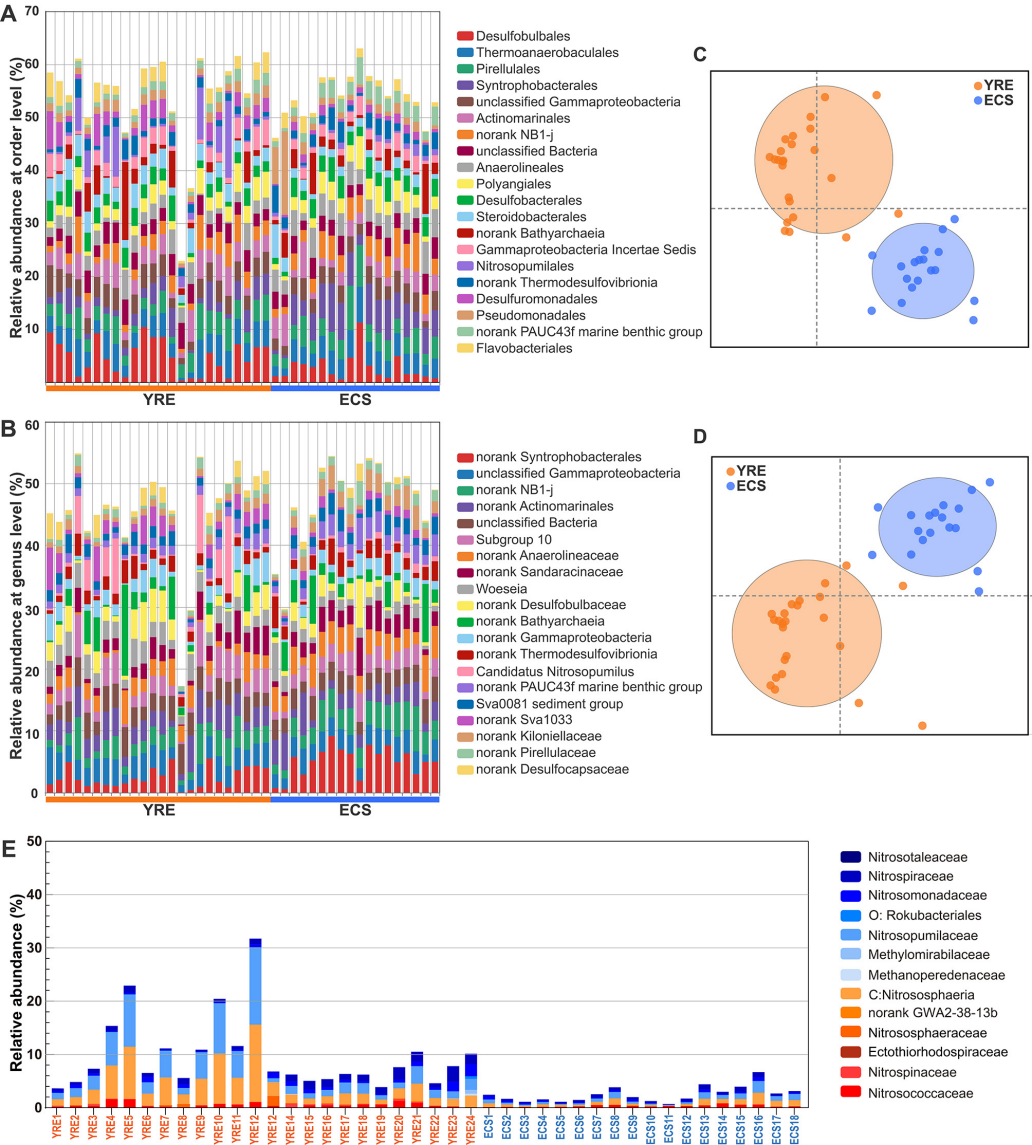
**(A, B)** Relative abundance of the most abundant microbes across sites at the order and genus levels, respectively. **(C, D)** Similarity of microbial community composition visualized by two-dimensional (2D) non-metric multidimensional scaling (NMDS) between the YRE and ECS at the order and genus levels, respectively. **(E)** Bar plots showing the relative abundance of microbial communities inferred to participate in nitrogen cycling (Mosley et al., 2022).

At the order level, the 10 most prevalent groups in the YRE were Desulfobulbales (4.85%), Thermoanaerobaculales (3.99%), Pirellulales (3.49%), Actinomarinales (3.35%), Steroidobacterales (2.93%), Desulfobacterales (2.61%), Polyangiales (2.54%), Anaerolineales (2.47%), Syntrophobacterales (2.46%), Desulfuromonadales (2.35%), and the 10 most prevalent groups in the ECS were Syntrophobacterales (5.12%), Pirellulales (3.85%), Thermoanaerobaculales (3.77%), Anaerolineales (3.77%), Polyangiales (2.96%), Desulfobacterales (2.91%), Actinomarinales (2.79%), Desulfobulbales (2.77%), Kiloniellales (2.48%), Pseudomonadales (2.38%; Figure 4A).

Non-metric multidimensional scaling (NMDS) analysis based on different taxonomic levels showed clearly distinguished microbial communities in the YRE from those in the ECS (Figures 3G, 4, Supplementary Dataset 2J; at phylum levels: PERMANOVA pseudo *F* = 4.0882, *P* = 0.002, 999 permutations; ANOSIM *P* = 0.008, 999 permutations). Further, partial distance-based redundancy analysis using environmental indicators as constraining variables further indicated that human activities, especially those altering water quality and chemistry, best explained the microbial community dissimilarity between the YRE and ECS (Figure 3H).

### 3.4 Community assembly processes in sediments

**Figure 5:**
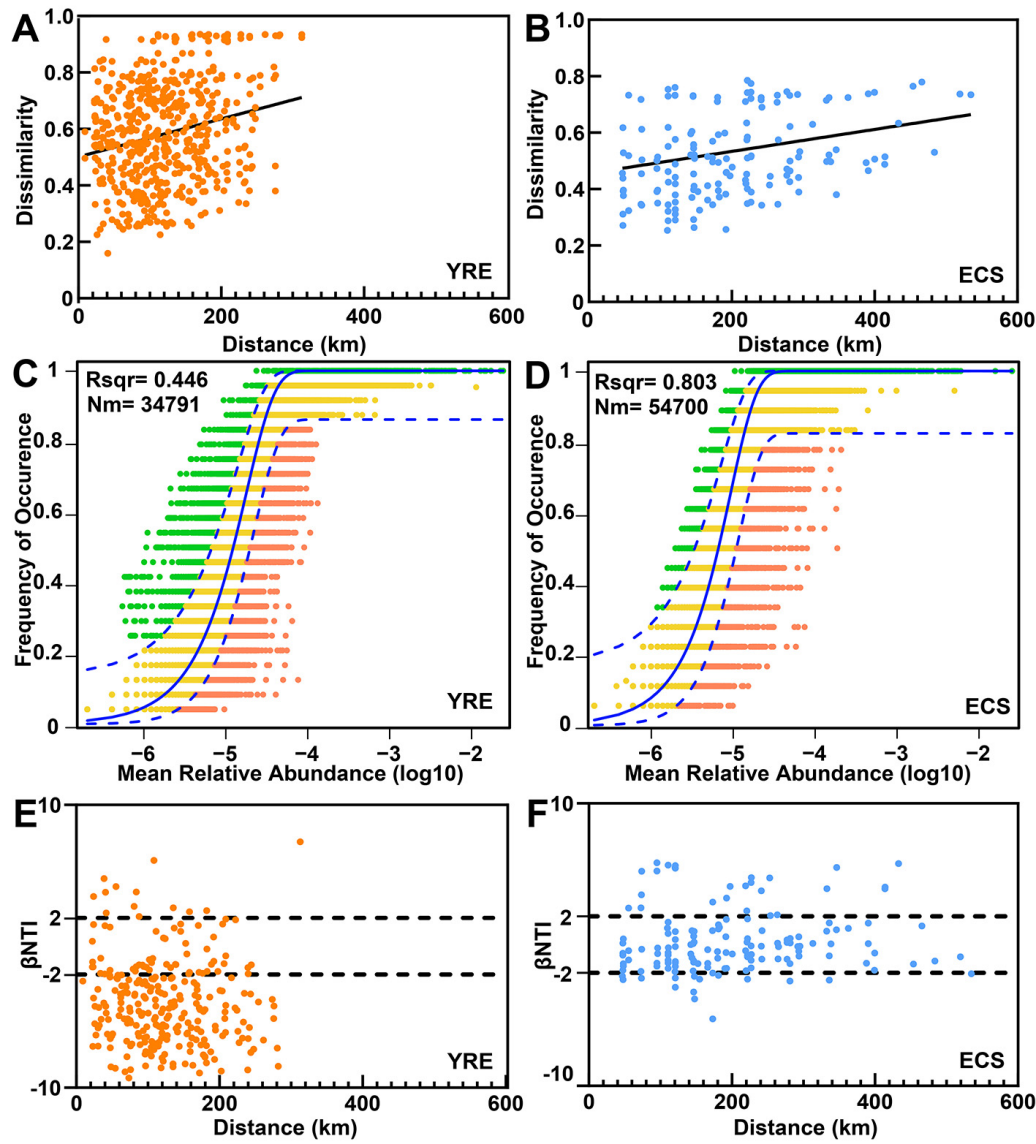
**(A, B)** Distance–decay curves depicting the relationship between Bray–Curtis similarity and geographical distances among sampling sites. The solid lines represent ordinary least-squares linear regressions. **(C, D)** The fit of the neutral community model (NCM) for community assembly is presented for the YRE and ECS. The solid blue lines indicate the best fit to the NCM, as described by Sloan et al., whereas the dashed blue lines represent the 95% confidence intervals around the model predictions. OTUs that occur more or less frequently than those predicted by the NCM are highlighted in different colors. Nm represents the metacommunity size multiplied by immigration, and *R*^2^ represents the fit to this model. **(E, F)** Variations in microbial communities explained by deterministic processes (such as homogeneous or heterogeneous selection) and stochastic processes (such as dispersal limitation) were compared between the YRE and ECS sediments on the basis of null model analysis.

Compared to the ECS, the YRE exhibited a steeper distance–decay relationship in microbial community composition (Figures 5A, B), indicating a higher rate of spatial turnover. To disentangle the ecological processes underlying community assembly, we applied both the neutral community model (NCM) and null model framework.

According to the NCM results, stochastic processes had a greater explanatory power in the ECS (accounting for 80.3% of community variation) than in the YRE (44.6%; Figures 5C, D). This decline in stochasticity in the YRE correlated with increased anthropogenic impact. The estimated Nm values, which serve as proxies for microbial dispersal potential, were notably higher in the ECS (*Nm* = 54,700) than in the YRE (*Nm* = 34,791), further supporting the dominance of neutral processes in the ECS.

However, since the NCM could not fully explain the observed community variation, we employed a null model to further dissect the contributions of deterministic vs. stochastic drivers. Results from the null model indicated a stronger influence of deterministic selection in the YRE (Figures 5E, F). Notably, there was a marked shift toward homogeneous selection in highly eutrophic YRE sites (βNTI <−2), reflecting strong eutrophication effects (Figures 5E, F).

Moreover, the variation partitioning analysis (VPA) and Mantel/partial Mantel tests consistently reveal contrasting microbial community assembly mechanisms in the YRE and ECS. In the eutrophic YRE, environmental factors explained a larger proportion of community variation (36%) than spatial factors (17%), with 12% jointly explained, indicating dominant environmental filtering (Supplementary Figure S2). This pattern is supported by Mantel tests, where microbial community dissimilarity was strongly correlated with environmental factors (*r* = 0.68, *P* = 0.001), and partial Mantel tests controlling for spatial effects remained significant (*r* = 0.64, *P* = 0.001). Partial Mantel tests based on Spearman rank correlation further confirmed that multiple environmental factors, particularly ammonia, nitrate, phosphate, chlorophyll a, and dissolved oxygen, were significantly associated with community dissimilarity. In contrast, in the oligotrophic ECS, both environmental (12%) and spatial (18%) effects were weaker, while a high residual (64%) suggests stochastic assembly. Mantel and partial Mantel tests showed low and non-significant correlations for both environmental and spatial factors (*r* = 0.12–0.15, *P* > 0.1). Partial Mantel tests using Spearman rank correlation indicated only weak associations with a few factors, such as silicate, dissolved oxygen, nitrate, and salinity, suggesting that random processes, including ecological drift and dispersal limitation, primarily shape ECS microbial communities.

### 3.5 Microbial life-history traits and their functional genes in sediments

**Figure 6:**
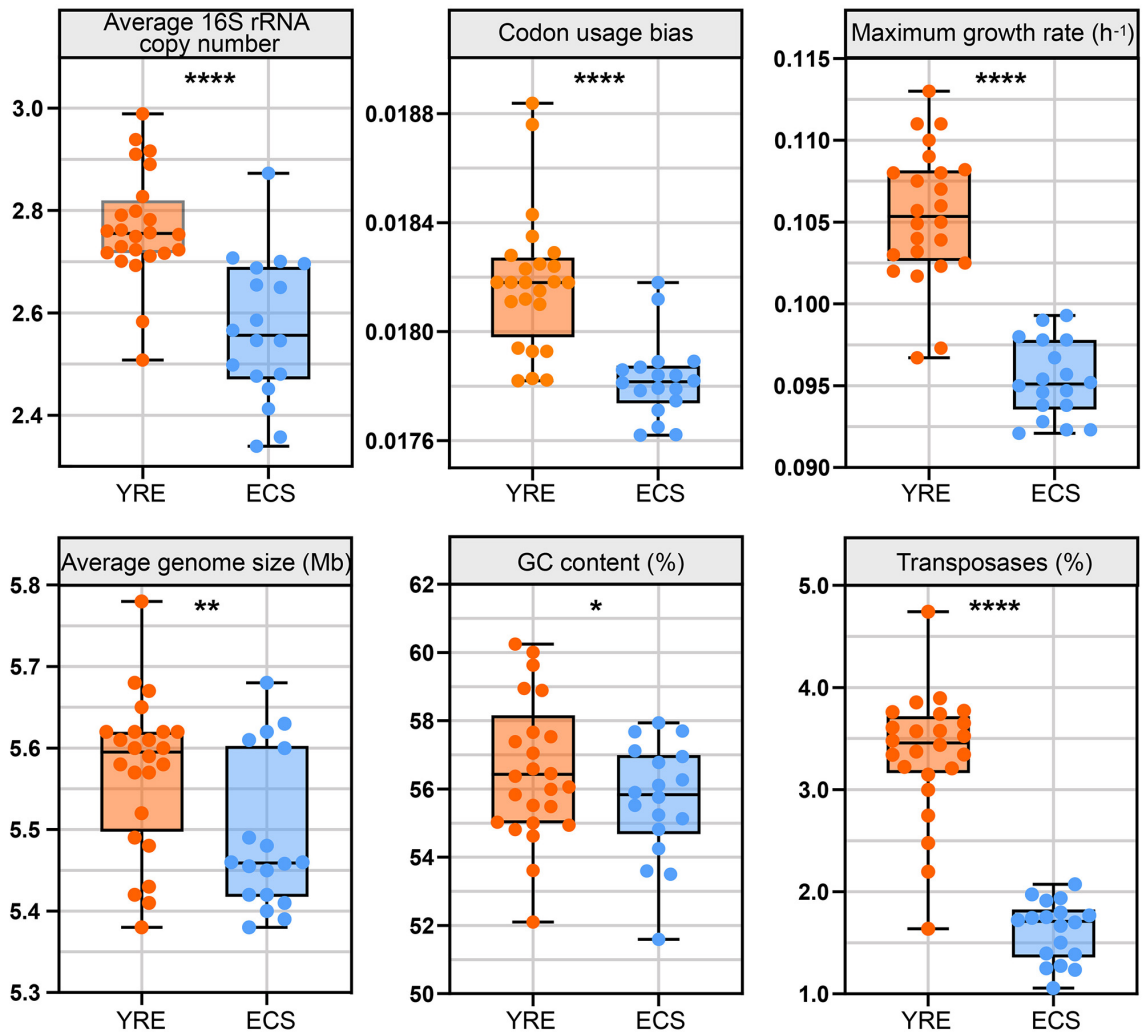
Box plots illustrating the comparison of life-history trait values between the YRE and ECS. The medians for these traits in the box plots are as follows (YRE vs. ECS): average 16S rRNA copy number (2.75 vs. 2.56), codon usage bias (0.0181 vs. 0.0178), maximum growth rate (0.1054 h^−1^ vs. 0.0951 h^−1^), average genome size (5.59 vs. 5.46 Mb), GC content (56.43 vs. 55.83%), and transposase content (3.46 vs. 1.71%). Sample sizes are *n* = 24 for YRE and *n* = 18 for ECS. Statistical comparisons were performed using the Wilcoxon rank-sum test with FDR correction (Benjamini–Hochberg method) and asterisks indicate significant differences between YRE and ECS samples (*P < 0.05; **P < 0.01; ****P < 0.0001).

To investigate microbial life-history traits and functional profiles, we conducted metagenomic sequencing on 42 sediment samples, generating a total of 1008.98 Gb of raw data, averaging 24.02 Gb per sample (Supplementary Dataset 3A). Based on the metagenomic data, we calculated several microbial life-history traits, including the average 16S rRNA gene copy number, codon usage bias in ribosomal genes, maximum predicted growth rate, genome size, GC content, and transposase abundance (Figure 6).

The average 16S rRNA gene copy number was significantly higher in the YRE (median = 2.75) than in the ECS (median = 2.56), implying a greater prevalence of fast-growing, resource-responsive taxa in the estuarine sediments. Codon usage bias (CUB), an indicator of translational efficiency, was significantly higher in the YRE (median = 0.0181) compared to the ECS (median = 0.0178), which may reflect stronger selection for rapid protein synthesis in more nutrient-rich conditions. The maximum predicted growth rate, estimated from codon usage in ribosomal genes, also showed higher values in the YRE (median = 0.1054 h^−1^) than in the ECS (median = 0.0951 h^−1^), consistent with copiotrophic traits and fast-growing organisms prevailing in the eutrophic YRE. Moreover, average genome size in the YRE (median = 5.59 Mb) was larger than in the ECS (median = 5.46 Mb), potentially reflecting a broader functional repertoire required to cope with variable or anthropogenically influenced conditions. GC content was also higher in the YRE (median = 56.43%) compared to the ECS (median = 55.83%), which may relate to genome stability under environmental stress. Notably, transposase gene abundance was markedly higher in the YRE (median = 3.46%) than in the ECS (median = 1.71%), indicating increased genome plasticity and potential horizontal gene transfer activity, likely driven by higher nutrient loads and environmental disturbances in the estuarine region. Overall, these results highlight a shift toward *r*-strategy life-history traits in the YRE microbial community under eutrophication pressure.

**Figure 7:**
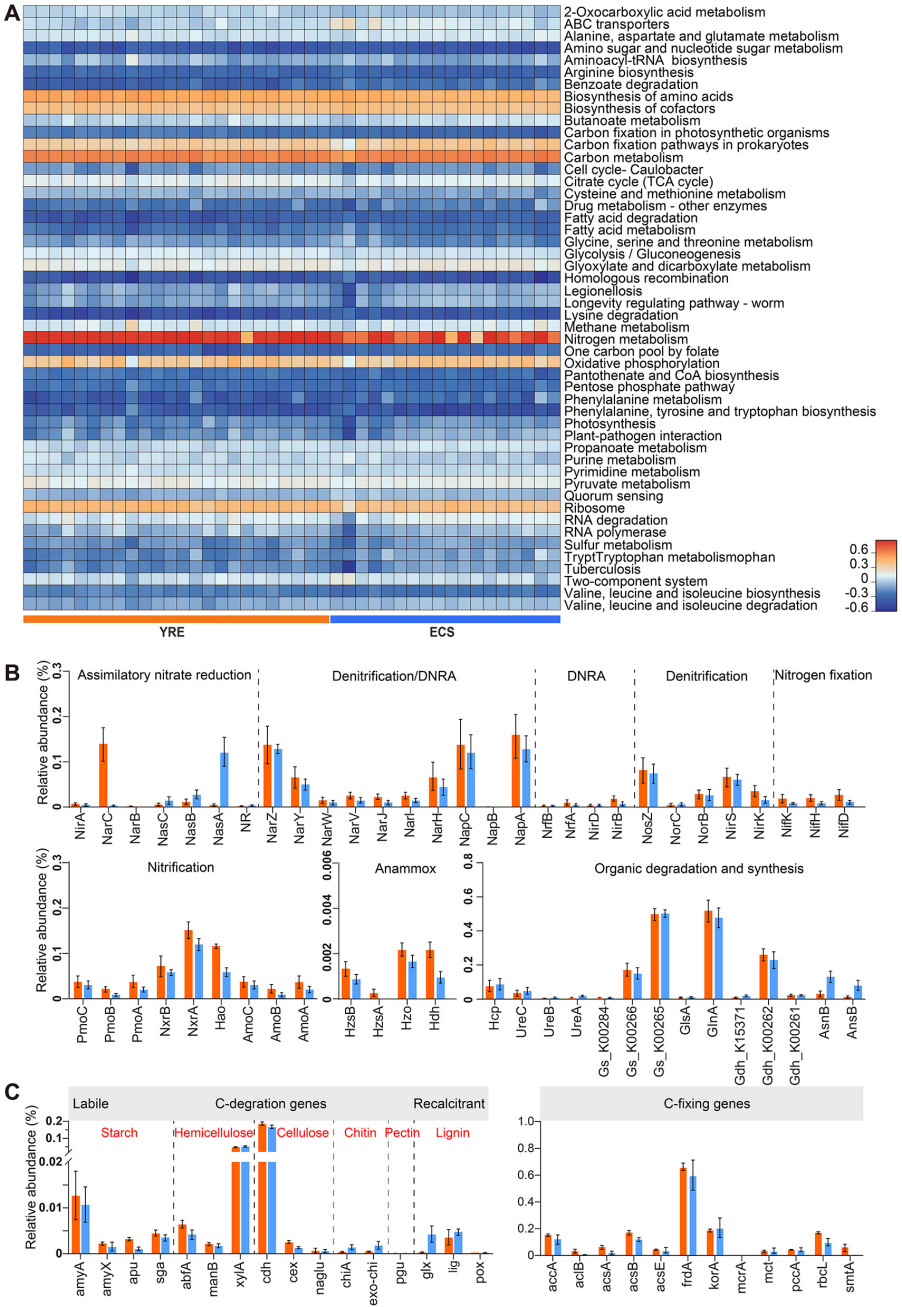
**(A)** Heatmap of the top 50 enriched KEGG pathway annotations. **(B)** Bar plots illustrating the relative abundance of sequence reads encoding dissimilatory and assimilatory nitrogen-cycling proteins based on metagenomic data, expressed as a proportion of all nitrogen-cycling processes. The orange bar plots represent YRE samples, and the blue bar plots represent ECS samples. Amo, ammonia monooxygenase; Pmo, particulate methane monooxygenase; Hao, hydroxylamine oxidoreductase; Nxr, nitrite oxidoreductase; Nar, nitrate reductase (dissimilatory); Nas, nitrate reductase (assimilatory); NirK, copper-containing nitrite reductase; NirS, cytochrome cd1-containing nitrite reductase; Nor, nitric oxide reductase; Nos, nitrous oxide reductase; Nif, nitrogenase (various); Hcp, hydroxylamine reductase; Nir, NADPH-nitrite reductase; Nrf, nitrate reductase (associated with Nap); Hdh, hydrazine hydrogenase; Hzs, hydrazine synthase; Hzo, hydrazine oxidoreductase. **(C)** Relative abundance of C degradation genes and C fixation genes. The genes are arranged according to the biodegradability of their target substrates, from labile to recalcitrant. amy, α-amylase; apu, amylopullulanase; sga, endoglucanase; abfA, α-L-arabinofuranosidase; manB, β-mannosidase; xylA, xylanase; cdh, cellobiose dehydrogenase; cex, cellobiohydrolase; naglu, β-glucosidase; chiA, endochitinase; exo-chi, exochitinase; pgu, polygalacturonase; glx, glyoxal oxidase; lig, laccase; mnp, manganese peroxidase; pox, lignin peroxidase; acc, acetyl-CoA carboxylase carboxyl transferase; acl, ATP-citrate lyase; acs, acetyl-CoA synthetase; frd, fumarate reductase; kor, α-ketoglutarate oxidoreductase; mcr, methyl-coenzyme M reductase; mct, malonyl-CoA transacylase; pcc, propionyl-CoA carboxylase; rbc, ribulose-bisphosphate carboxylase; smt, nickel transporter.

Functional pathway profiling of the microbial communities was performed using KEGG annotations (Figure 7A; Supplementary Datasets 3C, D). The dominant pathways included “Nitrogen metabolism” (13.99%), followed by “Carbon metabolism” (8.26%), “Biosynthesis of amino acids” (5.2%), and “Ribosome” (5.17%; Figure 7A, Supplementary Dataset 3D).

On the basis of Nitrogen pathway profiles, a total of 56 nitrogen-cycle gene (sub)families were detected using NCycDB (Tu et al., 2018), encompassing nitrogen fixation, anammox, nitrification, denitrification, dissimilatory nitrate reduction to ammonium (DNRA), ammonia assimilation, and organic nitrogen mineralization (Figure 7B, Supplementary Dataset 3E). While these gene families were present in both YRE and ECS samples, their relative abundances varied significantly.

In the YRE, genes related to ammonia oxidation (amo, hao), nitrite oxidation (nxr), and nitrate reduction (nar) were significantly enriched (*P* < 0.05, Wilcoxon Rank-Sum Test). Likewise, DNRA-associated genes (nrf), denitrification genes (nir) and anammox were more prevalent (*P* < 0.05, Wilcoxon Rank-Sum Test), indicating a more active nitrogen-removal potential under eutrophic estuarine conditions. In contrast, the ECS showed significantly greater abundance of nasA (*P* < 0.05, Wilcoxon Rank-Sum Test), a gene involved in assimilatory nitrate reduction. ECS also exhibited enrichment in nitrogen-storage-related genes, such as those encoding L-asparaginase II (ansB) and asparagine synthetase (asnB), suggesting a microbial strategy for nitrogen conservation under nutrient-limited conditions (Figure 2).

Analysis of carbon metabolism gene families also revealed distinct functional patterns between regions (Figure 7C). In the YRE, we observed increased abundance of genes encoding carbohydrate-degrading enzymes—such as α-amylase (amyA), glucoamylase (amyX), hemicellulase (abfA), and amylopectinase (apu)—which may reflect an adaptation to elevated organic matter inputs. By contrast, the ECS samples showed enhanced glyoxal oxidase (glx) activity, associated with lignin breakdown.

## 4 Discussion

Marine sediments serve not only as dynamic sites for carbon and nitrogen cycling but also as long-term sinks for both terrigenous and aquatic pollutants (Bao et al., 2024). Thus, elucidating how waterborne contaminants influence the diversity and functional attributes of sedimentary microbial communities is of critical ecological importance (Chen J. et al., 2019). In this study, geochemical indicators derived from comprehensive monitoring of the YRE and ECS revealed a clear spatial gradient in water mass influence. Specifically, the spread of nutrient-rich Yangtze Diluted Water (YDW) was confined to west of 123°E, whereas oligotrophic ECS waters dominated to the east, effectively limiting YDW's eastward penetration (Figures 1, 2; Liu et al., 2021). This sharp transition across a relatively narrow spatial scale offers an ideal natural setting to explore microbial responses to varying levels of anthropogenic disturbance in sedimentary environments.

### 4.1 Estuarine eutrophication alters microbial diversities and community compositions in sediments

Microbial profiling revealed that the YRE sediments harbored significantly greater microbial abundance, taxonomic richness, alpha diversity, niche breadth, and phylogenetic distance than their ECS counterparts (Figures 3A–E). These findings are consistent with the “productivity/resource hypothesis,” which posits that greater resource availability and/or primary production can support larger microbial populations, thereby facilitating species coexistence (Ibarbalz et al., 2019). The YRE receives substantial terrestrial inputs of organic matter and nutrients, enhancing primary productivity and fueling microbial growth (Figure 2A). Moreover, terrestrial organic matter and phytoplankton-derived compounds—rich in labile carbohydrates and amino acids—are readily bioavailable substrates for microbial metabolism (Moran et al., 2022; Battin et al., 2016). Consistent with this, functional profiling indicated that microbial communities in the YRE had a higher capacity to degrade labile carbon sources than those in the ECS (Figure 7C). Hence, eutrophic estuarine conditions can enhance both microbial abundance and diversity.

In highly impacted areas of the YRE, we detected significant enrichment of microbial taxa typically associated with terrestrial and gastrointestinal environments, including Actinobacteria (*P* < 0.01, Wilcoxon Rank-Sum Test), Firmicutes (*P* < 0.001, Wilcoxon Rank-Sum Test), and Bacteroidota (*P* < 0.001, Wilcoxon Rank-Sum Test; Supplementary Dataset 2C). Bacteroidota and Firmicutes are core constituents of the human gut microbiota (Chen J. et al., 2019; Ley et al., 2006). Actinobacteria are well-documented for resilience to environmental stressors and capacity to degrade pollutants such as pesticides, antibiotics, and heavy metals (Mohammadipanah and Wink, 2016). Although domestic sewage discharged into the Yangtze River is typically treated, the consistent detection of these phyla suggests residual anthropogenic influence. Conversely, Planctomycetota—a phylum frequently found in marine systems and considered a potential bioindicator for environmental quality—responds sensitively to elevated levels of heavy metals and inorganic nitrogen (Fuerst and Sagulenko, 2011). In our study, Planctomycetota ranked as the fourth most abundant phylum overall, but its relative abundance was significantly reduced in the YRE compared to the ECS (Figure 3F, Supplementary Dataset 2C), further indicating pollution stress in the YRE. Taken together, these patterns suggest that human activities—particularly those altering nutrient regimes and introducing contaminants—exert a profound influence on the microbial community compositions in estuarine systems (Figures 3G, H).

Moreover, nitrogen-cycling taxa exhibited a strong positive correlation with pollution levels, suggesting that eutrophication enhances the proliferation of microorganisms involved in nitrogen transformation (Figure 4E). Metagenomic analyses of sediment samples from both the YRE and ECS revealed that major nitrogen-cycling pathways—including ammonia oxidation, nitrification, denitrification, and dissimilatory nitrate reduction to ammonium (DNRA)—were broadly distributed across the study sites. However, their relative abundances differed markedly between the two regions (Figure 7B). Specifically, 36 out of 56 key nitrogen-cycling gene families were significantly more abundant in the YRE sediments than in the ECS (LEfSe analysis, Kruskal–Wallis test).

For instance, genes encoding ammonia monooxygenase (Amo) and hydroxylamine oxidoreductase (Hao)—enzymes catalyzing the rate-limiting steps of nitrification (Könneke et al., 2005)—were significantly enriched in the YRE, reflecting elevated activity of ammonia-oxidizing bacteria (AOB) and archaea (AOA; Figure 7B). This suggests that nitrogen-enriched estuarine conditions accelerate nitrogen cycling, potentially driven by inputs of terrigenous organic nitrogen and nitrate/nitrite that serve as favorable substrates for microbial metabolism. Similarly, the YRE also exhibited a higher prevalence of genes associated with DNRA, denitrification, and anaerobic ammonium oxidation (anammox), further emphasizing the enhanced nitrogen transformation capacity in eutrophic sediments. Microbial denitrification, while typically dependent on dissolved organic carbon availability, can also be fueled by reduced inorganic compounds such as iron and sulfur (Mosley et al., 2022). In the YRE, elevated concentrations of organic matter (Figure 2A), iron, and sulfur (Cao et al., 2015) may thus stimulate denitrifying microbial activity. Likewise, the presence of abundant organic matter and nitrite likely supports elevated DNRA and anammox processes (Yin et al., 2017). These functional profiles highlight the considerable potential for nitrogen removal in the YRE, particularly in oxygen minimum zones (OMZs; Figure 1). However, such processes may also generate undesirable byproducts, including nitrite—a toxic intermediate—and potent greenhouse gases such as nitric oxide and nitrous oxide, raising additional ecological concerns.

More direct evidence of anthropogenic influence on the estuarine microbiome emerged from analyses of putative pathogens (Supplementary Dataset 3G) and antibiotic resistance genes (ARGs; Supplementary Datasets 3H, I). The YRE sediments harbored significantly greater abundances of both pathogenic microbial taxa and ARGs (*P* < 0.0001 and *P* < 0.001, Wilcoxon Rank-Sum Test, respectively). Additionally, the YRE displayed a notably higher prevalence of transposase genes (Figure 6, Supplementary Dataset 3J), suggesting that enhanced horizontal gene transfer may facilitate the dissemination of ARGs among microbial populations. This is likely driven by strong selective pressures from antibiotic residues (Guo et al., 2019; Rizzo et al., 2013), alongside the nutrient-rich conditions that promote rapid microbial growth and high community density. Collectively, these findings indicate that intensive human activities are reshaping microbial community structure and function in estuarine sediments.

### 4.2 Estuarine eutrophication reshapes microbial community assembly and life-history strategies in sediments

Based on the above evidence, the YRE and ECS exhibited distinct taxonomic and functional profiles. However, pinpointing the precise ecological drivers remains challenging due to complex biotic–abiotic interactions and feedback mechanisms operating across temporal and spatial gradients (Martiny et al., 2011; Chen W. et al., 2019). In this study, the NCM and null model analyses revealed that ongoing eutrophication—characterized by elevated carbon and nitrogen inputs—has fostered specialized ecological niches in the YRE, leading to strong environmental filtering of microbial communities (Figure 3H). This environmental selection intensified the role of deterministic processes, particularly homogeneous selection, in shaping community assembly within the estuary (Figures 5C, E). In contrast, the ECS, with its relatively stable physicochemical conditions, lacked a strong environmental filter, resulting in weaker selection pressures and higher immigration rates (Figures 5D, F). As such, stochastic processes like ecological drift played a dominant role in driving the spatiotemporal variation of microbial abundances in ECS region.

To further explore microbial-environment interactions, we analyzed microbial life-history traits. Communities in the YRE displayed significantly higher average 16S rRNA gene copy numbers than those in the ECS (*P* < 0.0001, Wilcoxon Rank-Sum Test), indicating a higher cellular ribosome content in eutrophic environments (Ling et al., 2022). This trait is typically associated with copiotrophs, which possess high ribosomal content to support rapid responses to nutrient availability (Nemergut et al., 2015; Kearns and Shade, 2018). Moreover, the YRE communities exhibited significantly greater codon usage bias in ribosomal genes (*P* < 0.0001, Wilcoxon Rank-Sum Test), reflecting stronger selection for efficient translation during periods of rapid growth (Vieira-Silva and Rocha, 2010). Consequently, maximum predicted growth rates were significantly higher in the YRE than in the ECS (*P* < 0.0001, Wilcoxon Rank-Sum Test), suggesting a life-history strategy shift from *K*-strategists (adapted to nutrient-poor conditions) to *r*-strategists (adapted to nutrient-rich environments).

This *r*-strategist tendency in the YRE was further supported by functional gene profiles. Labile carbon-degrading genes, including α-amylase (amyA), glucoamylase (amyX), hemicellulase (abfA), and amylopectinase (apu), were enriched in the YRE, whereas genes associated with the degradation of recalcitrant carbon—such as endochitinase (chiA), polygalacturonase (pgu), and glyoxal oxidase (glx)—were more abundant in the ECS. This pattern reinforces the shift toward fast-growing, resource-opportunistic microbes in eutrophic estuarine environments.

Microbial communities in the YRE also exhibited a significantly higher average genomic GC content (Figure 6). According to the principles of nucleotide biosynthesis (Okie et al., 2020), GC base pairs require more nitrogen atoms (8 N) than AT pairs (7 N; Wang et al., 2024). The “resource-driven selection” hypothesis posits that nitrogen limitation favors lower GC content as a strategy to conserve nitrogen (Shenhav and Zeevi, 2020; Chuckran et al., 2023). The higher nitrogen levels in the YRE sediments likely contributed to nucleotide sequences with a G+C bias, supporting the notion of “resource-driven selection” in microbial communities, as they respond to nitrogen enrichment in estuarine ecosystems.

Compared with the YRE, microbial communities in the ECS exhibited significantly smaller average genome sizes (*P* < 0.01, Wilcoxon Rank-Sum Test). Many previous studies have shown that microorganisms in oligotrophic (nutrient-poor) environments often have relatively small genomes, with non-essential genes removed to conserve energy and resources (Giovannoni et al., 2014; Lauro et al., 2009; Lever et al., 2015). This reductive genomic evolution is explained by the Black Queen Hypothesis, which posits that gene loss, particularly of dispensable functions, can provide a selective advantage by conserving limited resources (Yin et al., 2024; Morris et al., 2012). In other words, from an evolutionary perspective, keeping genes that do not contribute to an organism's fitness is costly because of the burden on cellular processes and energetics (Yin et al., 2024). As a result, many microorganisms in oligotrophic oceans have evolved smaller, more streamlined genomes (Giovannoni et al., 2014), with microbes retaining only the genes essential for survival and growth. In contrast, the constant influx of organic matter, nitrogen, and phosphorus into YRE creates a dynamic ecosystem with fluctuating but consistently high nutrient levels (Figures 1, 2). Thus, to adapt to the variable estuarine environment, *r*-strategists possess larger genomes encoding a wide range of metabolic pathways, enabling them to respond rapidly to changes in resource availability and exploit diverse nutrient sources (Malik et al., 2019). As *r*-strategist microbes colonize and utilize a broad spectrum of resources, community-level niche breadth and phylogenetic distances expand (Figures 3D, E), indicating that YRE microbes are more likely to mutate and evolve than are natural ECS ecosystems.

It's worth noting that microorganisms under oligotrophic conditions exhibit more diverse genome sizes (Figure 6). In oligotrophic environments like the ECS, microbial genome size diversity can be greater than expected under purely streamlined life strategies. On one hand, many microbes in such settings evolve extremely small and streamlined genomes—such as *Pelagibacter ubique* and *Prochlorococcus* (~1–2 Mb)—to minimize energy and nutrient demands, a core prediction of streamlining theory aimed at maximizing growth efficiency under resource scarcity. On the other hand, the same ecosystem may harbor some larger-genome taxa, such as particle-associated or generalist microbes, that encode auxiliary metabolic pathways for episodic resource uptake or micro-niche versatility. The ecological coexistence of both genome extremes contributes to the broader genome size variance observed in ECS communities. Similarly, the low abundance of transposase genes in ECS microbial communities is consistent with selective pressure favoring metabolic efficiency and genomic stability. Transposases mediate horizontal gene transfer and genomic rearrangements, but maintaining mobile genetic elements imposes metabolic and regulatory costs. In resource-limited environments, natural selection tends to favor genomic stability and economy, leading to lower transposase densities (Zhong et al., 2024). Additionally, large-scale metagenomic surveys (e.g., Tara Oceans, Malaspina; Sunagawa et al., 2020; Duarte, 2015) show that transposase abundance correlates positively with greater genome sizes, which are more common in eutrophic, dynamic estuarine environments—contrasting with the stable, oligotrophic ECS niche where transposase prevalence remains low.

The trade-off between *r*- and *K*-strategies or copiotroph–oligotroph strategies, is a foundational concept in microbial ecology (Zhu and Dai, 2024). While *r*-strategies confers short-term advantages in resource-abundant environments, slow-growing *K*-strategies may exhibit superior longevity and stress tolerance (Jørgensen and Boetius, 2007). For instance, microorganisms in energy-limited deep marine sediments can persist for over 100 million years with minimal metabolic activity (Morono et al., 2020). Such observations underscore that *r*-strategists, despite their rapid growth, may be disadvantaged under long-term or stable conditions, where *K*-strategists exhibit superior survival traits (Lennon and Jones, 2011; Moger-Reischer and Lennon, 2019; Jones and Lennon, 2010; Starnawski et al., 2017). Therefore, while eutrophication enhances the growth potential of microbial communities in YRE, it may also shift the community composition toward opportunistic traits at the expense of stress-tolerant strategies. This shift could potentially reduce the ecological stability and resistance to future disturbances.

Although this study provides important insights into the effects of eutrophication on estuarine microbial communities, it is limited to a single sampling timepoint. Seasonal fluctuations in temperature, nutrient inputs, hydrodynamics, and biological interactions are well-known to influence microbial community structure and function in coastal and estuarine systems. The patterns observed here may be influenced by seasonal conditions and may not fully represent annual variability. Therefore, we acknowledge that our study lacks long-term observational data. Future studies incorporating multi-seasonal and multi-year sampling will be essential to confirm the temporal stability of the microbial responses documented in this work. Additionally, integrating time-series sampling with controlled nutrient amendment experiments and mesocosm studies would provide a more mechanistic understanding of how temporal environmental variability interacts with eutrophication to shape microbial life-history strategies and ecosystem resilience. Despite this limitation, the current study provides a valuable baseline and framework for understanding microbial ecological responses along eutrophication gradients in estuarine environments.

## 5 Conclusion

Taken together, our findings underscore the pervasive influence of eutrophication on the taxonomic composition, functional potential, and life-history strategies of estuarine microbial communities. Nutrient enrichment in the YRE favors fast-growing, metabolically flexible populations, potentially reducing the representation of slow-growing taxa associated with ecological stability and persistence. These community shifts may affect the ability of microbial assemblages to buffer environmental fluctuations and sustain critical biogeochemical processes. These findings highlight the importance of proactive nutrient management strategies, particularly in major watersheds like the Yangtze River Basin, to mitigate eutrophication pressures and preserve the ecological integrity of coastal and estuarine ecosystems. We advocate for integrated nutrient management strategies—such as reducing agricultural runoff and improving wastewater treatment—as essential measures to mitigate eutrophication pressures.

## Data Availability

The 16S rRNA amplicon and metagenomic sequencing data have been deposited in the NCBI Sequence Read Archive under accession numbers PRJNA1198359 and PRJNA1218293, respectively.
