# Supplementary material for: Eutrophication shifts microbial communities and life-history strategies in the Yangtze River Estuary
Source: Front Microbiol. 2025 Sep 15;16:1650511. doi: 10.3389/fmicb.2025.1650511 (PMC12493985; doi:10.3389/fmicb.2025.1650511)
Supplement: Supplementary file 5 [file Supplementary_file_1.docx]

Supplementary materials for

**Eutrophication shifts microbial communities and life-history strategies in the Yangtze River Estuary**

Haizhou Li^1,2 *^, Feng Zhao^1^, Qunhui Yang^2^, Lang Chen^1^, Jin Zhou^1, *^

Email: lihaizhou@ecsf.ac.cn; zhou_jin@foxmail.com

1. East China Sea Fisheries Research Institute, Chinese Academy of Fishery Science, Shanghai, China.

2. State Key Laboratory of Marine Geology, Tongji University, Shanghai, China.

A
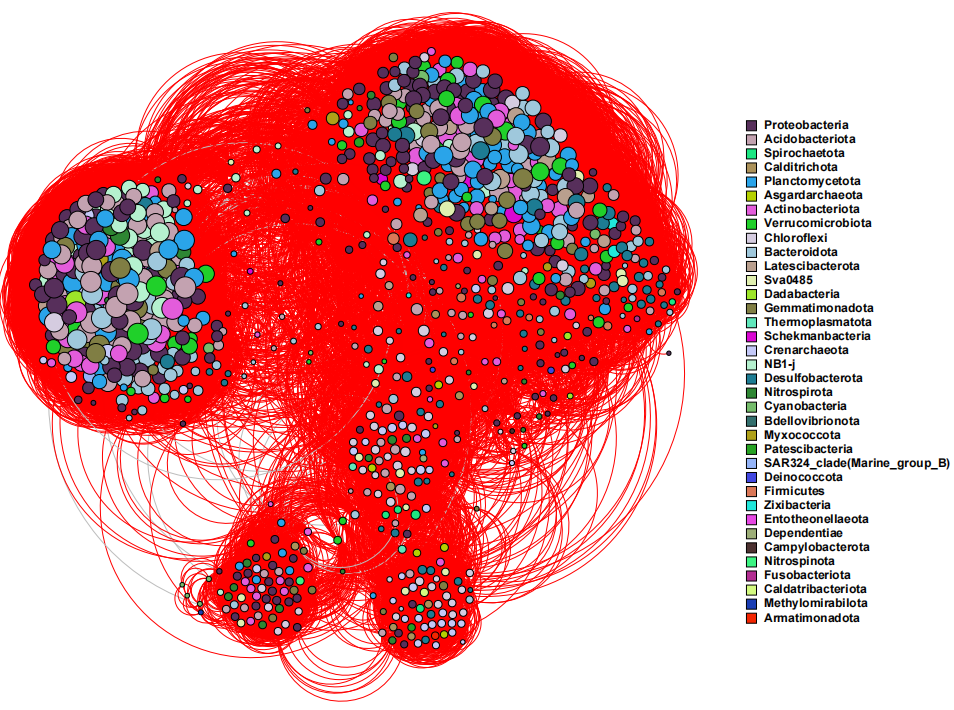


B
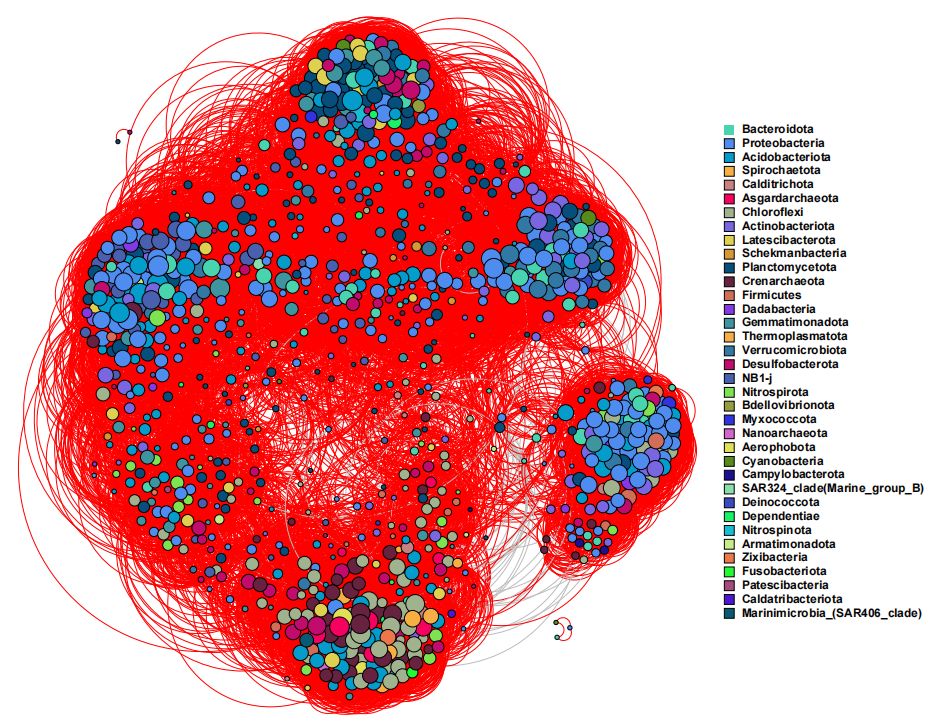


**Fig. S1** Co-occurrence networks in sediment microbiomes in YRE (A) and ECS (B). The co-occurrence networks of the microbial community based on pairwise Spearman’s correlations between OTUs. A connection indicates a strong (Spearman’s ρ > 0.6) and significant (FDR-corrected *P* < 0.01) correlation. The size of each node is proportional to the number of connections.


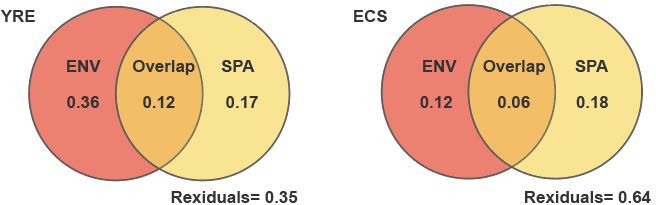


**Fig. S2** Variation partitioning analysis performed to quantify the contribution of environmental (ENV) and spatial (SPA) factors to community variations in YRE and ECS.
